# Green growth as a determinant of ecological footprint: Do ICT diffusion, environmental innovation, and natural resources matter?

**DOI:** 10.1371/journal.pone.0287715

**Published:** 2023-09-13

**Authors:** Ali Hassan, Juan Yang, Ahmed Usman, Ahmer Bilal, Sana Ullah

**Affiliations:** 1 Department of Media and Communication Studies, The Islamia University of Bahawalpur, Bahawalpur, Pakistan; 2 Chinese Academy of Science and Technology for Development, Beijing, China; 3 Department of Economics, Government College University Faisalabad, Faisalabad, Pakistan; 4 School of Economics, Zhongnan University of Economics and Law, Wuhan, China; 5 School of Economics, Quaid-i-Azam University, Islamabad, Pakistan; Kwame Nkrumah University of Science and Technology, GHANA

## Abstract

The nexus between green growth and ecological footprint is associated with crucial environmental implications. But this domain is not examined sufficiently and provides ambiguous findings. Furthermore, these studies have not addressed the role of natural resources, environmental innovation, and ICT in influencing ecological footprint. Our study analyzes the impact of green growth, ICT, environmental innovation, and natural resources on the ecological footprint ofemerging-7 and developed-7 economies. We employed CS-ARDL methodology to draw the long-run and short-run estimates of the said relationships. The obtained findings show that green growth, ICT, and environmental innovation reduce the ecological footprint in emerging economies in the long run. However, natural resources enhance the ecological footprint in emerging economies in the long run. Green growth, ICT, natural resources, and environmental innovation reduce the ecological footprint in the long run in developed economies. Based on these outcomes, the study recommends important policy suggestions.

## Introduction

Governments are embracing the "green growth" discourse in more and more nations to emphasize and promote their goals for the sustainability of their economy. The core principle of this narrative is that the furtherance of environmental sustainability creates economic opportunities rather than challenges. While there are numerous definitions of green growth, we will use the frequently cited OECD definition of green growth as "fostering economic growth and development while ensuring that natural assets continue to provide the resources and environmental services on which our well-being depends." Hence, green growth is proposed as a key component in achieving sustainable development: on the one hand, conserving the environment while allowing economic growth on the other hand. This may make the concept more appealing to politicians and other decision-makers than traditional environmental protection approaches, which were frequently assumed to result in an economic recession. The use of green growth for the sustainability of economic progress and, subsequently, for achieving sustainable development has become an important policy objective for most of the world’s governments, including E7 ((Brazil, Russia, India, China, South Africa, Mexico, and Indonesia) and G7 (Canada, France, Germany, Italy, Japan, the United Kingdom, and the United States) economies [1].

In recent years, both the E7 and G7 countries have shown a growing interest in promoting green growth to achieve sustainable development. This has been driven by the recognition that traditional economic growth models that rely on exploiting natural resources are unsustainable in the long run and threaten the environment [2]. The G7 countries have adopted policies to reduce greenhouse gas emissions, promote renewable energy, and encourage the adoption of sustainable practices by businesses and individuals [3]. For example, Canada has implemented a carbon pricing scheme to encourage a shift towards low-carbon energy sources, while Germany has committed to phasing out nuclear power and increasing the share of renewable energy in its energy mix [4]. The G7 countries have also been at the forefront of international efforts to address climate change and promote sustainable development, as seen in the Paris Agreement on climate change and the Sustainable Development Goals. On the other side, the E7 countries have focused on promoting green growth to achieve sustainable development and reduce poverty rather than as an end in itself [2]. For example, China has invested heavily in renewable energy and has set targets for reducing carbon intensity, but it continues to rely heavily on coal for its energy needs [5]. India has also made significant progress in promoting renewable energy but faces challenges in balancing economic growth and environmental sustainability [6]. While both the G7 and E7 countries are committed to promoting green growth, their approaches and priorities differ significantly. The G7 countries have been able to adopt more ambitious policies due to their high levels of economic development, while the E7 countries have placed a greater emphasis on achieving economic growth and development. Irrespective of their policy differentials, both groups of countries have an essential role in promoting sustainable development and addressing the global challenges of climate change and environmental degradation. Hence, exploring the factors influencing climate change in both G7 and E7 nations is crucial. Green growth is one such factor that helps improve environmental quality in both groups of countries and drives them on the path of sustainability.

Another important research query this analysis addresses is whether environmental innovation, ICT diffusion, and natural resources complement green growth in achieving superior environmental standards for both E7 and G7 economies. Developing products and processes using new ideas, concepts, and technologies while considering their economic and environmental effects is called eco-innovation [7]. Green technologies are recommended to become the fundamental basis of a truly innovative technological revolution, and industrial management in the development of green industry segments so they help preserve long-term economic expansion and highly paid jobs [8]. Innovations in the production of energy while using the latest environment-friendly technologies can be fruitful for improving environmental quality [9]. It can also effectively reduce the carbon footprints on our ecological system, create efficient energy, and conserve the world’s natural resources [10]. The role of green technologies in reducing wastage and trash during production and consumption activities is beyond doubt, and thus their function is mandatory in promoting green practices in society [11].

Economies have observed enormous productivity and energy efficiency due to the utilization of ICT over the last three decades. However, the effects of excessive use of ICT in multi-domain on the ecological system remained neglected, resulting in its footprint on the ecological system and environmental sustainability remaining inconclusive [12]. Studies on the use of ICT have shown mixed results; few of them encourage the use of ICT as it reduces the carbon footprint; however, few claim its use harms the environment [13, 14]. Likewise, reducing production costs, increasing resource efficiency, and greater investments can be connected with ICT diffusion [15]. However, higher electricity has been consumed by ICT infrastructure; subsequently, more amount of carbon emissions was observed at the global level same is evident from electricity consumption figures from 2007 to 2012, which are 3.9% to 4.6% respectively [16]. Therefore, a significant negative impact is also visible on our ecological system after the rapid growth of ICT [17]. At the same time, as per U.N. Climate Change Conference 2015, using ICT can reduce the carbon emission footprint on our ecological system by 20%. Environmental pollution can be controlled in a befitting manner by efficient use of ICTs instead of unnecessary use of transport, reduction in manpower employment, and environment-friendly technologies [18].

Disproportionate use of natural resources since the 1980s has contributed heavily to climate change and global warming issues, resulting in a critical threat to accomplishing sustainable development goals. The relationship between natural resources and environmental deterioration is a contentious issue. On one side, economic expansion and related urbanization and industrialization developments increase the need for the exploitation and use of national resources, which has an unsustainable impact on the ecosystem [19]. Conversely, an excess of natural resources might deter the use of fossil fuels by lowering imports [20]. Sarkodie [21] also argues that human actions, such as mining and deforestation, are some of the main contributors to the degradation of natural habitats as well as water, soil, and air pollution. The empirical examination of how natural resources contribute to environmental quality came to identical conclusions as these arguments. For example, Hassan et al. [22] find that using natural resources causes the ecological footprint to rise, although other research claims the contrary.

In recent times, significant development has occurred in the environmental literature, where empirics have started preferring ECF over CO2 emissions as a measure of environmental degradation. The term "ecological footprint" describes the total impact of human activities, which may be estimated by considering factors such as the proximity of bio-efficient land, the quantity of water needed to produce food, and the amount of waste generated. It may have represented the environmental impact caused by manufacturing necessities for a middle-class lifestyle [23]. As ECF considers both direct and indirect influences affecting the ecosystem, it is gaining popularity as an indicator of environmental damage in recent empirical investigations [21].

Even though ECF is widely acknowledged as a preferred measure of environmental degradation compared to CO2 emissions; however, the studies that have used ECF to represent environmental degradation are still at their beginning stage. In addition, green growth should be the way to achieve environmental and economic sustainability, but no past study has examined the link between green growth and ECF; particularly, comparative analysis in the context of E7 and G7 economies is missing the current body of environmental literature. Further, the current body of literature does not clearly define the role of eco-innovations, natural resources, and ICT in determining environmental quality and provides inconclusive results.

The primary objective of the current analysis is to close the above-stated gaps in the literature by analyzing the impact of green growth, ICT diffusion, eco-innovations, and natural resources on ECF in G7 and E7 economies by utilizing the STIRPAT model. Collectively these countries account for one-third of global production and are among the top polluted footprint economies. The E7 has made significant progress over the last 20 years, which has allowed them to catch up to the G7. Nonetheless, because of their expanding energy needs and such a high development trajectory, the E7 is vulnerable to environmental problems. The E7 countries are likely to become the key player by 2050 [24], but they need to invest more in improving their environmental standards. Compared to E7, the G7 are the world’s top advanced economy. The G7 has implemented strict ecological laws to address ecological damage in order to lessen reliance on fossil fuels and address the issue of climate change. Despite the rigorous efforts put in by both sets of nations for the world’s sustainable future, the issue of environmental sustainability is still a great menace for this and upcoming generations. Since green growth is gaining popularity as a panacea to environmental problems; hence, it is crucial to estimate the impact of green growth on environmental quality in E7 and G7.

Against this backdrop, this study is a vital addition to the current environmental economics literature and thus makes the following contribution. First, to our limited knowledge, this is the first effort to investigate the impact of green growth on ECF using the STIRPAT model of G7 and E7 economies. Second, in addition to green growth, we have also included ICT, eco-innovations, and natural resources as a determinant of ECF, which have been used separately in past studies but not collectively. Third, this analysis used ECF instead of CO2 emissions to represent environmental degradation. Fourth, most past studies suffered from various methodological issues, such as cross-sectional dependence, slope heterogeneity, etc. To overcome these issues, we have employed the latest panel data methods to perform the analysis, including the Cross-Sectionally Augmented Autoregressive Distributive Lag Order (CS-ARDL, for baseline regress estimation) and Dynamic Common Correlated Effects (DCCE, for robustness check) of Chudik and Pesaran [25]. Fifth, this is the first study to investigate the nexus between green growth, natural resources, eco-innovations, ICT, and ECF in the context of G7 and E7 economies. Last but not least, policymakers in both developed and developing economies may benefit greatly from the policy suggestions offered by this research, which may prove vital for the world’s sustainable development.

## Literature review

Numerous research using various estimating strategies, such as panel data analysis and time series analysis, have been motivated by the issue of environmental deterioration. Nevertheless, a large collection of research has relied on CO2 emissions as a measurement of environmental degradation and ignored the ecological footprint. Measuring the size of the ecological footprint is the major focus of the most available literature. Recently, researchers looked at the other factors that impact ecological footprint. In this section, in addition to the studies using CO2 emissions, we have also reviewed the studies using ecological footprint. This study has covered the literature that has attempted to disentangle the connections between environmental sustainability and green growth, eco-innovation, information and communication technology, and natural resources.

### Green growth and environment

In the wake of the worldwide financial crisis of 2008, a collection of studies on green growth has appeared, with much of it arising from the successful implementation of such strategies in affluent nations. So far, the work on green growth has not only covered basic theoretical and analytical concepts but also has covered, but is not confined to, the following topics: employment, technology and research, and trade. Since green growth can meet both ecological preservation and economic progress, it is especially important for attaining sustainable development. Green growth can provide substantial social and economic advantages since it is seen as efficient and cost-effective and vital for the survival of under-developing regions (Development, O. for E.C, 2013). Resource efficiency, valuation of natural capital assets in economic calculations, energy system transformation, and the price of externalities attached to environmental degradation are all things that may be gained through enacting green development strategies [26]. Resource efficiency, valuation of natural capital assets in economic calculations, energy system transformation, and the price of externalities attached to environmental degradation are all things that may be gained through enacting green development strategies [26]. Most nations are committed to green growth in their development priorities, but not all follow the same precise plan. More and more studies are being conducted on CO2 and environmental protection, including the many facets of sustainable practices and their interplay with economic, ecological, and political factors. More and more studies are being conducted on CO2 and environmental protection, including the many facets of sustainable practices and their interplay with economic, ecological, and political factors. However, consultancies and foreign donor organizations are researching green growth and exploring how green growth can impact different economic activities. Though only a small number of research articles have directly addressed green growth, every one of those that has done so has used it a left hand side variable [11].

### Eco-innovations and environment

As the world economy is currently on the brink of witnessing the "Fourth Industrial Revolution", technological advancement is regarded as the principal factor for reaching the Sustainable development goal [27]. In this regard, technological improvements are anticipated to affect environmental characteristics [28]. Moreover, eco-innovation is seen to be the remedy for the world’s ecological concerns [29]. In the past, CO2 emissions have been employed as an indicator of environmental deterioration to anticipate the relationship between eco-innovation and environmental performance. Using data from G7 nations, Wang et al. [30] explore the relationship between eco-innovation and carbon footprint and conclude that eco-innovation alleviates ecological damage by lowering CO2 discharges. Similarly, Ding et al. [31] said that eco-innovation was instrumental in lowering the G7 nations’ consumer-driven CO2 emissions. Several studies have also observed the positive role of eco-innovations in improving environmental quality, such as Zhang et al. [32] for China, Solarin and Bello [33] for the United States and Hashmi and Alam [34] for OECD countries.

Connecting technology advancements to environmental protection in developing countries in both the short- and long-term, Ahmad et al. [27] show that technological improvements diminish the ECF. In addition, scholars claim that technical breakthroughs and ECF are interdependent. Likewise, Gormus and Aydin [35] prove that technological advancements reduce the long-run ECF concentrations in a sample of chosen OECD countries. In addition, the country-specific data demonstrate that technical improvements effectively reduce the ECF in Finland, Korea, and the United States. Usman and Hammar [36] find that technology developments decrease environmental quality in APEC member states. Consequently, it is possible to assert that the environmental effects of technological breakthroughs are often equivocal.

### Natural resources and environment

There are a variety of viewpoints on whether or not a country’s wealth may be directly tied to its natural resource wealth. Even though some researchers claim that an excess of natural resources boosts economic expansion in resource-abundant countries by enhancing trade and output, others assert that it actually hinders development in the long term because these natural assets eventually run out [37]. Wang et al. [38] investigate China’s "resource curse" concept. Their result demonstrates the reality of the "resource curse" in China. They also show that investments in technology and people boost economies.

Many studies in current years have focused on natural resources and environmental degradation. Some researchers have looked at how different types of natural resources affect CO2 production, and they fall into the first category. For example, Balsalobre-Lorente et al. [20] examine the correlation between GDP and carbon footprints in the EU-5 by controlling the effects of natural resources. They point to an inverse relationship between pollutants and natural resources and an N-shaped relationship between affluence and pollutants.

Likewise, Joshua and Bekun [39] find that resource rent helps lower pollution in South Africa. Furthermore, environmental pollution results from economic expansion and coal use. In comparison, Danish et al. [19] employ the AMG technique to examine the relationship between emissions, GDP, and renewables in the BRICS. The AMG-based panel assessment shows that natural resources have a negligible impact on CO2 emissions, EKC exists between GDP and CO2 emissions, and renewable energy negatively influences emissions.

The second group of studies includes ECF as a proxy for environmental degradation. For instance, natural resources reduce ECF in the U. S., according to research by Zafar et al. [40]. Human capital has also shown a positive correlation with ECF, whereas energy use and household income negatively impact environmental quality. Danish et al. [19] show that the BRICS countries have a similar connection between natural assets and ECF. The EKC also appears to exist in BRICS nations, and it is shown that the use of clean energy and urbanization may lower ECF.

On the other hand, Hassan et al. [22] claim that although human capital has no significant influence on ECF; however, natural resources and urbanization lower ECF in Pakistan. They also demonstrate the existence of EKC in Pakistan. Generally speaking, there is little research on this issue, and the importance of natural endowments appears to alter among nations. It is clear that the administration and exploitation methods for natural resources determine whether they have harmful or beneficial consequences.

### ICT and environment

Literature on the impact of ICT and environmental quality is on the rise. Again, most authors have utilized CO2 emissions to measure environmental degradation. According to Asongu et al. [41], ICT may be utilized to lessen the possible harm that pollution could have to human evolution. In order to investigate the impact of clean and dirty energy resources, GDP, financial development, and ICT on ecological performance in the top 10 polluting counties, Caglar et al. [42] employed ARDL-PMG. They demonstrate that the expansion of ICT, financial progress, and clean energy lowers environmental quality.

Nevertheless, other researchers have shown that the manufacturing and usage of ICTs cause environmental harm [43]. For instance, Lee and Brahmasrene [17] utilized the panel cointegration approach to show how ICT increased carbon pollution in ASEAN countries between 1991 and 2009. The relationship between aggregate productivity, CO2 emissions, and ICT in Tunisia from 1975 to 2014 is also examined by Amri et al. [18]. The ARDL finding indicates that ICT has no noticeable effect on CO2 emissions.

Recently, some authors have investigated the relationship between ECF and ICT. For instance, Caglar et al. [42] gathered data for the top ECF economies and observed the positive impact of ICT in restricting ECF. On the other side, in the context of Saudi Arabia, Kahouli et al. [44] suggested the insignificant relationship between ICT and ECF in the long run, while the negative link between ICT and ECF is confirmed in the short run. Khan and Ximei [45] investigate the link between ICT trade and ECF in G7 economies. The results of the study suggest that ICT export enhances ECF; however, ICT import reduces ECF in G7 economies.

In light of the above-stated literature, we can point out the following gaps in the literature:

1. In most past studies, empirics have tried to find the determinants of green growth and used it as a dependent variable, while in this study, we used it as a determinant of environmental quality.
2. Most past studies have used CO2 emissions as a proxy of the environment, while the studies that have used ECF to represent environmental quality are still in its infancy stage.
3. The role of natural resources, eco-innovations, and ICT in affecting environmental quality is not clearly defined.
4. The majority of research that looked at this link in different circumstances had methodological issues.
5. No study has analyzed the impact of green growth on ECF in the context of advanced and emerging economies.
6. In the context of comparative research between the G7 and E7 economies, no study has looked at the impacts of green growth, natural resources, eco-innovations, and ICT on ECF. This research closes this gap as a result.

## Data, model, and methods

### Data

The primary aim of this study is to estimate the impact of green growth, ICT, natural resources, and eco-innovations on the ecological footprints of G-7 and E-7 economies over the period 1995–2020. The G-7 or advanced economies include France, Canada, Japan, Italy, Germany, the USA, and the UK. In contrast, the E-7 or emerging economies include Brazil, China, Mexico, Indonesia, Russia, India, and Turkey. In this analysis, we have used ecological footprint instead of CO2 emissions to represent environmental degradation. Ecological footprint refers to the measure of the impact of human activities on the environment. Data on ecological footprint is gathered from a global footprint network. Our independent variables include green economic growth and conventional economic growth, natural resources, eco-innovations, ICT, energy consumption, and population. Green growth refers to the development of an economy that is environmentally sustainable, socially inclusive, and economically resilient. It aims to promote economic growth while reducing greenhouse gas emissions, preserving natural resources, and enhancing social well-being [46]. In this study, it is measured by environmentally adjusted multifactor productivity growth. ICT diffusion refers to the spread of information and communication technology (ICT) across different regions and populations. It includes the adoption and use of technologies such as computers, mobile phones, the internet, and other digital devices [47]. The level of ICT diffusion is often measured by indicators such as the number of internet users, mobile phone penetration rates, and the number of households with computers or broadband access. Our study measures ICT diffusion through the number of internet users as % of the population. Eco-innovation refers to the development and implementation of new technologies, processes, and products that have a positive impact on the environment. The level of eco-innovation is often measured by indicators such as the number of patents filed for eco-friendly technologies, the amount of investment in clean technology, and the adoption of sustainable business practices by firms [48]. In this study, eco-innovation is measured by environment-related technologies as % of all technologies. Conventional growth involves the production and consumption of goods and services, which are often associated with high levels of environmental degradation and carbon emissions [49]. In our study, it is measured by real gross domestic product per capita. Energy consumption includes the consumption of both non-renewable and renewable sources of energy, such as oil, gas, coal, hydropower, wind, and solar [50]. Energy consumption in this study is measured by kg of oil equivalent per capita. Population refers to the number of individuals living in a particular area or country. Population is measured by total number of people in thousands. Natural resources is measured by total natural resource rent as % of GDP. Data on green growth and eco-innovations are gathered from the OECD, and data on conventional economic growth, natural resources, ICT, energy consumption, and population are collected from the world development indicators. In Table 1, we’ve laid out all the variables, their respective sources, and their respective measurement units to benefit our readers.

**Table 1:** Variables and sources.

Variable	Symbol	Unit	Source
Ecological footprint	ECF	Global hectares per capita	Global Footprint Network
Green growth	G.G.	Environmentally adjusted multifactor productivity growth	Organization of Economic Cooperation and Development
ICT diffusion	ICT	Individuals using the Internet (% of population)	World Development Indicator
Eco-innovations	EIN	Environment-related technology, % of all technologies	Organization of Economic Cooperation and Development
Conventional growth	GDP	Real Gross Domestic Product Per Capita (Constant 2010 $ U.S.)	World Development Indicator
Energy consumption	EC	kg of oil equivalent per capita	World Development Indicator
Population	POP	Total (in thousands)	World Development Indicator
Natural resources	NR	Total natural resource rent (% of GDP)	World Development Indicator

### Model

The most prominent approach for understanding how human activities influence the ecosystem is IPAT identification. In the early 1970s, Holdren and Ehrlich [51] introduced the IPAT classification initially. Ever since, it has been an essential starting point for determining the primary causes of environmental concerns. IPAT has various limitations despite being a flexible and economical approach that can rapidly identify the main reasons for climate change. For example, the principal environmental factors’ non-monotonic or non-proportional effects are disregarded by the IPAT framework. Dietz and Rosa [52] introduced STIRPAT, which refers to "stochastic (S.T.) impacts (I) through regression (R) on population (P), affluence (A), and technology (T)". STIRPAT is a more advanced IPAT identity with every one of the benefits of the IPAT structure without any downsides.

In recent times, the use of the STIRPAT model in studying the relationship between environmental quality and its determinant has increased manifold. For instance, Liddle [53] extended the model of ICT with renewable energy, Abou-Ali et al. [54] with government effectiveness, Lin et al. [55] with urbanization and industrialization, Yang et al. [56] with trade openness, Dedeoğlu et al. [57] with immigration and human capital, and Usman et al. [58] with nuclear energy, among others. In light of the available literature, we have augmented the STIRPAT model by including eco-innovations and ICT as technology variables and natural resources and energy consumption as additional variables. Hence, the econometric model we constructed is as follows:

$$LN{ECF}_{it}=\alpha_{0}+\alpha_{1}{LNPOP}_{it}+\alpha_{2}LN{GDP}_{it}+\alpha_{3}{LNICT}_{it}+\alpha_{4}LN{ECO}_{it}+\alpha_{5}LN{NR}_{it}+\alpha_{6}{LNEC}_{it}+\varepsilon_{it}$$ (1)

Where ECF stands for ecological footprint, which is based on total population (POP), affluence or per capita income (GDP), information and communication technology (ICT), environmental-related innovations or eco-innovations (ECO), natural resources (NR), electricity consumption (EC), and error term (ε). In order to resolve the normalcy issues in the data, we have taken the natural log of all variables [19].

The primary motive of the analysis is to see the influence of green growth on environmental quality in G7 and E7. Green growth, commonly referred to as eco-friendly economic progress, is multifactor productivity that has been adjusted for the environment. It has recently been seen as an important tactic for achieving a sustainable economy and environment. Classical ideas of economic growth call for the consumption of more natural capital, which contributes to the problem of environmental destruction. As a result, officials everywhere are putting increasing emphasis on green development, which is more durable and cleaner than conventional growth. Modern growth concepts have also highlighted the need for the economy to undergo a green transition since the current strategy progressively degrades the environment [59, 60]. According to Jacobs [8], the fundamental purpose of the concept of "green growth" is to accomplish economic objectives without disturbing the earth’s ecological balance. Consequently, we have modified the STIRPAT model and included green economic growth as an indicator of affluence instead of conventional economic growth, as shown below:

$$LN{ECF}_{it}=\beta_{0}+\beta_{1}{LNPOP}_{it}+\beta_{2}LN{G.G.}_{it}+\beta_{3}{LNICT}_{it}+\beta_{4}LN{ECO}_{it}+\beta_{5}LN{NR}_{it}+\beta_{6}{LNEC}_{it}+\varepsilon_{it}$$ (2)

In the above Eq (2), we have incorporated green growth (G.G.) as a representative of affluence, while the rest of the variables are the same as already described previously in Eq (1).

### Methods

In this study, the number of observations varies across T & N, referred to as panel data settings. Our data set consists of 26 years (T) and two separate sets of 7 cross-sections (N), suggesting that T > N. As a result, we choose to use the panel cointegration test. Traditional approaches like the fixed and random effects models are more appropriate when N > T; however, in the dataset where T > N, panel cointegration techniques are more appropriate [61]. Although panel data provides many benefits, most recent research has indicated that Cross-Sectional Dependence (CSD) in residuals is the main drawback of panel data. We live in a world where countries are interdependent and economically connected, leading to CSD [62]. For checking CSD, we use various CSD test details, which are given in the next section. Prior to examining the cointegration of our variables, finding a variable’s unit root attributes is crucial. Unit root tests must be used in order to prevent bogus regression. Second-generation unit root test, such as CIPS advanced by Pesaran [63], is appropriate to examine the variables’ stationarity in the presence of CSD and slope heterogeneity.

In order to check the validity of the long-run connection between the variables, we need to perform cointegration tests. The Westerlund [64] ECM panel cointegration test can yield reliable outcomes, regardless of the presence of the CSD. Westerlund [64] is advantageous since it allows for several structural breaks in the panel series and uses the approach developed by Bai and Perron [65] to identify the break position endogenously.

After checking CSD, stationary, and cointegration, we believe that the CS-ARDL model is the most appropriate method because it can deal with CSD and handle the blend of I(0) and I(1) variables. The CS-ARDL model of Chudik and Pesaran [25] is an augmentation of the conventional panel ARDL-PMG [66] that we used to assess the relationship between our variables. A short-run and long-run cross-sectional average of each relevant variable are included in the CS-ARDL structure together with short-run and long-run parameters, error correction terms, and other components. There are many benefits of this approach over others. First, it can still produce reliable estimates even when the variables are included in different ordering, like I(0) or I(1). Then, it can provide correct findings when both short-run and long-run CSD occur [25].

Moreover, it is a mean group estimate with diverse slope coefficients. The CS-ARDL dependent on the mean group is an enhanced version of the ARDL method that uses average cross-sectional estimates for each cross-section as a proxy for common unobserved variables and their lags. Finally, this methodology is effective when a weak exogentiy arises from the model’s lagged dependent variable. Hence, based on the following specification, we have constructed our baseline regression model:

$$\Delta {ECF}_{it}=C_{i}+\lambda_{i}({ECF}_{it-1}-\beta_{i}X_{it-1}-\phi_{1i}{\bar{ECF}}_{t-1}-\phi_{2}{\bar{X}}_{t-1})+\sum_{j=1}^{p-1}\;\theta_{ij}\Delta {ECF}_{it-j}+\sum_{j=0}^{q-1}\;\zeta_{ij}\Delta X_{it-j}+\eta_{1i}\Delta {\bar{ECF}}_{t}+\eta_{2i}\Delta {\bar{X}}_{t}+\varepsilon_{it}$$ (3)

Where the dependent variable ∆ECF is dependent on a set of independent variables represented by X_it_ in the long run; ${\bar{ECF}}_{t-1}$ represent the long-run average of the dependent variable and ${\bar{X}}_{t-1}$ represent the long-run average of independent variables; in the short run, Δ*ECF_it−j_* signifies a dependent variable, while Δ*X_it−j_* represents a collection of explanatory variables; $\Delta {\bar{ECF}}_{t}\&\Delta {\bar{X}}_{t},$ represent the short-run averages of dependent and independent variables; *ε_it_*, is an error term that is randomly distributed. In addition, j stands for the units of the cross-section, t for time, and β_i_ for the long-run coefficients of the variables that are considered independent; *θ_ij_*, stands for the short-run coefficient attached to the dependent variable and *ζ_ij_*, for the short-run coefficient attached to the independent variable; *η*_1*i*_&*η*_2*i*_ coefficients are attached to the short-run mean values of dependent and independent variables. Lastly, we apply the panel Granger causality test created by Dumitrescu and Hurlin [67] to determine if our variables are causally related to one another. We favor this causality technique as it does not impose restrictions on T > N and delivers reliable findings in the situation of cross-sectional dependency. The null hypothesis of this test states that there is no causality within any of the cross-sections. The alternative suggests the existence of at least one causal link in the panel.

## Results and discussions

Results in Table 2 explain that the problem of cross-sectional dependence (CSD) mostly occurs during the estimations of large panel data. Moreover, if any disturbance happens in one nation, these waves affect other nations like a virus. Therefore, it is very important to limit CSD if we want to get unbiased estimates. Under this theory, we used Breusch-Pagan LM, Pesaran scaled LM, Bias-corrected scaled L.M., and Pesaran CD tests to see whether CSD is present in the data. According to our CSD estimates, CSD is present in our model.

**Table 2:** Results of CSD.

		Emerging Economies				Developed Economies		
	Traditional Growth		Green Growth		Traditional Growth		Green Growth	
	Statistics	Prob.	Statistics	Prob.	Statistics	Prob.	Statistics	Prob.
Breusch-Pagan LM	108.556***	0.000	134.281***	0.000	88.291***	0.000	104.78***	0.000
Pesaran scaled LM	13.510***	0.000	17.479***	0.000	10.383***	0.000	12.928***	0.000
Bias-corrected scaled LM	13.376***	0.000	17.345***	0.000	10.249***	0.000	12.793***	0.000
Pesaran CD	-3.875***	0.000	-3.858***	0.000	-3.911***	0.000	-3.808***	0.000

In Table 3, we tested the slope heterogeneity test [68] and observed that we firmly accept the alternate hypotheses stating that the heterogeneity problem is present in both our emerging and developed economies cases. In a more simplified form, these findings indicate that the model’s coefficients are also diverse and that the slope differs among nations. This also shows that other nations cannot directly influence the social and economic structures of any other nation [69]. Also, this suggests that we should expect erroneous interference in the form of skewed or confused findings if we try to impose homogeneity limits on the study results that are generated from the panel data [70]. Due to the existence of CSD and heterogeneity in our data, we need to employ the second-generation unit root test.

**Table 3:** Slope heterogeneity.

		Emerging Economies			Developed Economies			
	Traditional Growth		Green Growth		Traditional Growth		Green Growth	
	Statistics	Prob.	Statistics	Prob.	Statistics	Prob.	Statistics	Prob.
Delta	12.015	0.00	8.8	0.00	2.179	0.029	2.483	0.013
Delta-adj	15.019	0.00	9.013	0.00	2.724	0.006	3.103	0.002

Following preliminary tests that demonstrate Heterogeneity and CSD in the panel data, the next step is conducted to ensure that our variables are stationary, which will finally avoid the occurrence of any erroneous regression estimate [71]. For this particular purpose, we employ the CIPS unit root test. The CIPS test results are more reliable than other unit root tests because they are more accurate when CSD and heterogeneity factors are present. Table 4 shows that a few variables are I(0), i.e., stationary at level; however, some others are I(1), i.e., stationary at first difference both in developing and emerging economies. In our case, no variable is I(2).

**Table 4:** CIPS unit root.

	Emerging Economies			Developed Economies		
	Level	First-Difference	Decision	Level	First-Difference	Decision
LNEF	-2.55	-5.045***	I(1)	-2.76*	-4.972***	I(0)
LNGDP	-1.904	-3.599***	I(1)	-2.837*	-4.003***	I(0)
LNGG	-2.922**	-4.631***	I(0)	-4.838**	-6.031***	I(0)
LNNR	-2.211	-5.179***	I(1)	1.917	-3.915***	I(1)
LNICT	-6.177***	-7.682***	I(0)	-2.26	-4.153***	I(1)
LNEINO	-4.497***	-6.420***	I(0)	-3.818***	-5.465***	I(0)
LNEC	-2.118	-4.336***	I(1)	-3.859***	-6.290***	I(0)
LNPOP	-2.214	-4.192***	I(1)	-1.761	-4.052***	I(1)

⁎⁎⁎ represents 1%

** represents 5%

⁎ represents1% level of significance, respectively.

In the next step, after observing that our variables are in a mixed order of integration, we applied the Westerlund [64] test to see whether or not cointegration is present in our model. Results in Table 5 show that we strongly reject the null hypothesis of no-cointegration because the P-values of Gt and Pt are significant at the 1% level. In short, cointegration is present among the selected variables (LNECF, LNGG(LNGDP), LNNR, LNICT, LNEIN, LNEC, LNPOP) in both models. Verification of cointegration provides strong evidence for a long-term connection between the variables being investigated.

**Table 5:** Results of Westerlund co-integration test.

		Emerging Economies				Developed Economies		
	Traditional Growth		Green Growth		Traditional Growth		Green Growth	
	Statistics	Prob.	Statistics	Prob.	Statistics	Prob.	Statistics	Prob.
Gt	-3.323***	0.00	-2.923***	0.00	-3.395***	0.00	-2.933***	0.00
Ga	-2.482	1.00	-2.825	1.00	-6.394***	0.00	-4.216	1.00
Pt	-4.167***	0.00	-5.014***	0.00	-7.565***	0.00	-6.41***	0.00
Pa	-1.048	1.00	-1.171	1.00	-5.522	1.00	-3.655	1.00

⁎⁎⁎ represents 1%

** represents 5%

⁎ represents1% level of significance, respectively.

Table 6 presents the estimations of the CS-ARDL econometrics model. Our key focus will be on long-run estimates. However, we briefly discuss the short-run estimates for the readers’ interests. In the short run, the estimated coefficients of GDP per capita (∆LNGDP), natural resources (∆LNNR), energy consumption (∆LNEC), and population (∆LNPOP) imply that all these factors contribute to the ecological footprint in emerging economies in the traditional growth model, while eco-innovations (∆LNEIN) reduces ecological footprint. On the other side, the estimate attached to green growth (∆LNGG) is negatively significant in the green growth model, suggesting the positive role of GG in improving environmental quality by reducing ecological footprint in emerging economies, while the rest of the variables have the same sign and same interpretation. In the context of developed economies, the estimates of GDP per capita (∆LNGDP) and energy consumption (∆LNEC) are positive and significant, which signifies that these variables add to the ecological footprint and thus deteriorate environmental quality. In contrast, the negative estimates of natural resources (∆LNNR), ICT (∆LNICT), eco-innovations (∆LNEIN), and population (∆LNPOP) suggest that natural resources, ICT, eco-innovations, and population reduces ecological footprint in the short run. The negative estimate of green growth in the green growth model assured that green growth help reduces the ecological footprint in developed economies. The remaining variables have the same interpretation as done in the traditional growth model.

**Table 6:** Results of CS-ARDL.

		Emerging Economies				Developed Economies		
	Traditional Growth		Green Growth		Traditional Growth		Green Growth	
Variables	Statistics	T-stats	Statistics	T-stats	Statistics	T-stats	Statistics	T-stats
Long Run Estimates								
LNGDP	0.16**	2.14			0.07*	1.75		
LNGG			-0.04*	1.77			-0.06*	1.84
LNNR	0.03***	3.10	0.02*	1.76	-0.01***	3.35	-0.01*	1.66
LNICT	-0.02*	1.69	-0.02**	2.48	-0.03**	2.11	-0.01*	2.55
LNEIN	-0.01**	2.10	-0.07**	2.04	-0.02**	2.44	-0.03**	3.15
LNEC	0.10*	1.92	0.19*	1.84	0.52***	2.69	0.36***	4.51
LNPOP	0.03**	2.01	0.04**	0.05	-0.09***	2.51	-0.07*	1.93
Short Run Estimates								
∆ECT(-1)	-0.97***	-10.04	-0.95***	-8.85	-0.47***	6.01	-0.35***	3.49
∆LNGDP	0.29***	3.24			0.13***	4.32		
∆LNGG			-0.10*	1.74			0.09**	1.98
∆LNNR	0.05***	3.20	0.03*	1.83	-0.01***	3.42	-0.01*	1.76
∆LNICT	0.01	0.49	0.01	0.25	-0.04**	2.03	-0.02**	2.40
∆LNEIN	-0.01**	2.19	-0.02**	2.15	-0.03***	-3.60	-0.02***	2.35
∆LNEC	0.25*	1.72	0.41**	2.07	0.16***	2.86	0.23***	4.57
∆LNPOP	0.09***	3.06	0.13***	3.12	-0.57***	2.70	-0.38**	2.20
Constant	-4.39	-0.14	-1.49	-0.07	-3.79	0.13	-1.78	0.12

⁎⁎⁎ represents 1%

** represents 5%

⁎ represents1% level of significance, respectively.

Next, we will discuss the long-run estimates of traditional and green growth models in detail. In the traditional growth model of emerging economies, GDP per capita (LNGDP), natural resources (LNNR), energy consumption (LNEC), and population (LNPOP) coefficients display a very strong positive bonding with the ecological footprint. A 1% improvement in GDP, natural resources, energy consumption, and population increases ecological footprint by 0.16, 0.03, 0.10, and 0.03 percent, respectively. On the other side, estimated values of coefficients information and communication technology (LNICT) and eco-innovation (LNEIN) show a significant inverse impact on ecological footprint. A 1% increase in information and communication technology and eco-innovations improve the environmental quality by decreasing the ecological footprint by 0.02 and 0.01 percent.

In the green growth model of emerging economies, the estimates of LNGG, LNEIN, and LNICT adversely impact the ecological footprint. More precisely, if green growth, eco-innovations, ICT, natural resources, and population increase by 1%, it reduces the ecological footprint by 0.04, 0.07, and 0.02 percent. In contrast, estimates of LNNR, LNEC, and LNPOP show a statistically positive connection with the ecological footprint. A 1% rise in natural resources, energy consumption, and population size increase the ecological footprint by 0.02, 0.19, and 0.04, thus polluting the environment further.

In the case of developed economies, the coefficients of LNGDP and LNEC variables are significant and positive, while coefficient estimates of LNNR, LNICT, LNEIN, and LNPOP are negatively significant. Consequently, a 1% increase in GDP and energy consumption may increase the pollution in the environment by increasing the ecological footprint by 0.07 and 0.52 percent. In contrast, a 1% improvement in natural resources, ICT, economic innovation, and population cut the ecological footprint by 0.01, 0.03, 0.02, and 0.09 percent.

LNGG, LNNR, LNICT, LNEIN, and LNPOP estimates have a statistically significant negative connection with an ecological footprint in the green growth model. More specifically, a 1% evolution in green growth, natural resources, information and communication technology, economic innovations, and population reduces the ecological footprint and improves the environmental quality by 0.06, 0.01, 0.01, 0.03, and 0.07. Oppositely, the variable LNEC positively affects the ecological footprint. A 1% increase in energy usage contaminates the environment by increasing the ecological footprint by 0.36 percent, respectively.

Checking the robustness of the results has almost become a convention these days. Following the convention, we have employed DCCE introduced by Chudik and Pesaran [25]. This method accounts for heterogeneous slopes and CSD by including cross-sectional averages and lags. In addition, the jack-knife correction technique used in this procedure makes it suitable for use with small sample sizes [25]. Another great thing about this method is how well it handles estimates when there are structural gaps in the data [72]. Also, the results for imbalanced panel data are satisfactory when using this method [73]. Table 7 assesses the DCCE model explaining the long-run estimates. In emerging economies, the traditional growth model highlights that LNGDP, LNNR, LNEC, and LNPOP coefficients are statistically significant. A 1% rise in GDP, natural resources, energy consumption, and population surges ecological footprints by 0.53, 0.04, 0.45, and 0.08 percent, respectively. On the other side, the statistical values of LNICT and LNEIN illustrate a substantial converse influence on ecological footprint. Stating that a 1% growth in information and communication technology and economic innovations recover the environmental quality by diminishing the footprint by 0.01 and 0.02 percent.

**Table 7:** Results of DCCE.

		Emerging Economies				Developed Economies		
	Traditional Growth		Green Growth		Traditional Growth		Green Growth	
Variables	Statistics	T-stats.	Statistics	T-stats.	Statistics	T-stats.	Statistics	T-stats.
Long Run Estimates								
LNGDP	0.53**	2.03			0.16**	2.39		
LNGG			-0.10***	2.71			-0.25*	2.49
LNNR	0.04*	1.69	0.07**	2.31	-0.02*	1.92	-0.14	2.04
LNICT	-0.01***	3.31	-0.03**	2.19	-0.03**	2.02	-0.20	1.74
LNEIN	-0.02***	2.50	-0.02***	3.40	-0.05***	3.36	-0.01***	2.54
LNEC	0.45*	1.85	0.57**	2.43	0.88***	4.69	0.41***	6.03
LNPOP	0.08**	2.05	0.58***	2.74	-0.38**	2.45	-0.76	2.24
Constant	-65.31	-0.92	-21.88	-0.61	-5.27	0.14	11.79	0.46

⁎⁎⁎ represents 1%

** represents 5%

⁎ represents1% level of significance, respectively.

Furthermore, in the green growth model of emerging economies, regressors parameters LNGG, LNICT, and LNEIN negatively affect ecological footprint. A 1% increase in green growth, ICT, and economic innovation enlarged the footprint partially by 0.10, 0.03, and 0.02 percent. Contrariwise, variables of LNNR, LNEC, and LNPOP display a significant positive link with the ecological footprint. It means that a 1% upsurge in natural resources, energy consumption, and population causes the size of the ecological footprint to increase by 0.07, 0.57, and 0.58 percent and generate more trash into the environment.

In the context of developed economies, we confer that estimates of LNGD and LNEC are positively significant, and the estimates of LNNR, LNICT, LNEIN, and LNPOP are negatively significant in the traditional growth model. To be precise, a 1% increase in GDP per capita and energy consumption escalate the ecological footprint by 0.16 and 0.88 percent. Conversely, a 1% rise in natural resources, ICT, eco-innovations, and population significantly shrinks ecological footprint by 0.02, 0.03, 0.05, and 0.38 percent. In the green growth model, estimates of LNGG, LNNR, LNICT, LNEIN, and LNPOP are negative and significant, and the only estimate of LNEC is positive. In a specific form, a 1% improvement in green growth, natural resources, ICT, eco-innovations, and population reduces ecological footprint by 0.25, 0.14, 0.20, 0.01, and 0.76 percent and a 1% increase in energy consumption proliferates ecological footprint by 0.41%.

Table 8 shows the estimates of the D-H causality test. The causality test suggested bidirectional causality between LNEF and LNGDP, LNEF and LNGG, LNEF and LNICT, LNEF and LNEC, and LNEF and LNPOP in both emerging and developed economies. Similarly, bidirectional causality exists between LNEF and LNNR, and LNEF and LNEIN exist in developed economies. In comparison, unidirectional causality in emerging economies exists between LNEF and LNNR, LNEF and LNEIN.

**Table 8:** Results of D-H panel causality test.

		Emerging Economies		
Null Hypothesis:	W-Stat.	Zbar-Stat.	Prob.	Decision
LNGDP⇎ LNEF	5.09321	3.05730	0.0022	LNEF↔LNGDP
LNEF ⇎ LNGDP	5.66637	3.66765	0.0002	
LNNR ⇎ LNEF	3.07265	0.90562	0.3651	LNEF→LNNR
LNEF ⇎ LNNR	5.87956	3.89468	0.0001	
LNICT ⇎LNEF	3.98087	1.87277	0.0611	LNEF↔LNICT
LNEF ⇎ LN	5.88928	3.90503	9.00E-05	
LNEIN ⇎ LNEF	2.56403	0.36399	0.7159	LNEF→LNEIN
LNEF ⇎ LNEIN	3.95735	1.84773	0.0646	
LNEC ⇎ LNEF	4.17848	2.01831	0.0213	LNEF↔LNEC
LNEF ⇎ LNEC	3.42046	1.85379	0.08932	
LNPOP ⇎ LNEF	7.19696	5.29757	1.00E-07	LNEF↔LNPOP
LNEF ⇎ LNPOP	11.1517	9.50900	0.000	
LNGG ⇎ LNEF	4.31482	2.22839	0.0259	LNEF↔LNGG
LNEF ⇎ LNGG	5.88848	3.90418	9.00E-05	
	Developed Economies			
Null Hypothesis:	W-Stat.	Zbar-Stat.	Prob.	Decision
LNGDP⇎ LNEF	11.2814	9.64710	0.000	LNEF↔LNGDP
LNEF ⇎ LNGDP	7.81254	5.95310	3.00E-09	
LNNR ⇎ LNEF	6.19550	4.23112	2.00E-05	LNEF↔LNNR
LNEF ⇎ LNNR	4.60290	2.53517	0.0112	
LNICT ⇎LNEF	4.22578	2.13358	0.0329	LNEF↔LNICT
LNEF ⇎ LNICT	1.85469	-0.39138	0.6955	
LNEIN ⇎ LNEF	7.53157	5.65389	2.00E-08	LNEF↔LNEIN
LNEF ⇎ LNEIN	6.66043	4.72623	2.00E-06	
LNEC ⇎ LNEF	3.90918	1.9345	0.0413	LNEF↔LNEC
LNEF ⇎ LNEC	6.01133	4.03500	5.00E-05	
LNPOP ⇎ LNEF	3.98822	1.88060	0.06	LNEF↔LNPOP
LNEF ⇎ LNPOP	5.45355	3.44102	0.0006	
LNGG ⇎ LNEF	5.79360	3.80314	0.0001	LNGG↔LNEF
LNEF ⇎ LNGG	3.99918	1.93387	0.0404	

## Discussions

The key findings of the study confirm that conventional growth has increased the ecological footprint, and green growth has reduced the ecological footprint, meaning that conventional growth has deteriorated the environmental quality, while green growth has improved the environmental quality in advanced and emerging economies. As the economy expands, the energy demand also goes up. This is due to the fact that nations have started investing heavily in energy-intensive sectors, including industry, transportation, and agriculture. Moreover, economic activities such as consumption and production consume a huge amount of natural resources at the initial stages of development, seriously damaging the earth’s ecological balance and increasing the ecological footprint. Ecological problems may worsen when the economy moves from the low to the middle-income stage of development because economic growth degrades environmental quality [16, 74]. The positive impact of economic growth on environmental quality is also supported by the past findings of Huang et al. [75], Usman et al. [74], Yang et al. [56], and Lin et al. [55].

In comparison to the traditional notion of growth, green growth is more desirable for both developed and emerging economies due to its environmentally friendly nature. Our findings confirm the negative relationship between green growth and ecological footprint and are supported by the study of Jouvet and de Perthuis [26]. The concept of "green growth" promotes economic expansion without placing an additional strain on the world’s natural resources, which is necessary for a healthy ecosystem. According to Jacobs [8], green growth may help us simultaneously meet economic development and environmental conservation goals. Reilly [76] added that "economic development, job creation, and environmental impact reduction" are often the three main goals of green growth. Green growth is crucial because it encourages both ecological sustainability and economic growth. Green growth, which is widely seen as economically sound and crucial to the stability of advanced and emerging nations, might result in substantial social and economic benefits. These results may guide developed and developing nations to design pertinent strategies for a sustainable environment and economy. It is admirable that developed and developing nations are working toward environmental sustainability. The G7 and E7 nations do, however, make a significant absolute contribution to global warming emissions and also consume a lot of natural resources. Thus, developed and developing nations may lessen the strain on the ecosystem by implementing a plan for green development.

Next, natural resources play a conducive role in improving the environmental quality of advanced economies by reducing their ecological footprint. However, natural resources and ecological footprint are positively linked in emerging economies, implying that the rise in the consumption of natural resources degrades the environmental quality. The advanced technologies incorporating recyclable materials, remanufacture, innovation, value-adding, and synthetic resources replacing natural resources will promote economic expansion and improve environmental protection [19, 77]. Moreover, advanced economies ensure the sustainable use of natural resources due to their better institutional quality and management structure. Even though the G7 economies rely on imported fuels, the abundance of natural resources can help the countries replace the nonrenewable imported energy sources with alternative and renewable energy sources, fulfilling energy demand and reducing ecological footprint [78]. On the other side, the positive relationship between natural resources in emerging economies is because extraction methods are outdated and utilization of natural resources is not sustainable, which generates more waste, thereby increasing the ecological footprint in E7 economies. The exploitation of natural resources includes mining and deforestation, which may result in a rising ecological footprint. Additionally, the excessive use of natural resources in emerging economies, such as coal, petroleum, and natural gas, to fulfill their energy demand causes environmental degradation [22, 27].

Our findings also confirm that ICT can help improve environmental quality in developed and emerging economies. If ICT adoption boosts production efficiency, environmental protection may be upheld even while a country is producing more and growing rapidly [79]. The concoction of production, input, and technology effects of ICT outweigh the scale effect of ICT, and consequently, the negative impact of ICT on ecological footprint is shown [12, 14]. Further, ICT is crucial in transforming the economy into digitalized and dematerialized one due to replacing physical resources with information resources [74]. Consequently, the economy achieves the status of a weightless economy. Thus, large-scale ICT penetration in society helps lower the ecological footprint and has a favorable effect on the environment in industrialized and emerging nations. It also implies that ICT can separate development from environmental degradation [80].

Further, eco-innovations improve environmental quality in advanced and developing nations, supported by past studies such as Afrifa et al. [81] and Tao et al. [82]. The fundamental concept of eco-innovations is the creation of unique goods, processes, and techniques that must consider economic and ecological sustainability and, as a result, produce fewer waste materials during manufacturing and consuming actions [7]. Additionally, eco-innovations are created to manage trash and use natural assets efficiently as possible, which is essential for protecting the environment [32, 83]. Many industrialized and emerging nations have raised the role of eco-technologies in their pollution control strategy because of the efficacy of these technologies in conversing with the ecosystem [30, 84].

In the context of emerging economies, the relationship between population and ecological footprint is positive, suggesting that an increase in population causes the deterioration in environmental quality. Previously, the studies of Sadorsky [85], Salim et al. [86] also observed similar types of results for emerging and developing economies. Along with the increasing population, the demand for several factors, such as urbanization, transportation, housing, and energy consumption, also increases [87], negatively impacting air and water quality, agriculture grounds, and forest zones, thus increasing the ecological footprint [88]. In contrast, the population increase reduces the ecological footprint in advanced economies. According to Usman et al. [58], the people of advanced economies are far better educated than that of emerging economies, which may raise environmental consciousness among the population. Accordingly, the desire for a clean environment increases in nations with higher incomes. Moreover, highly educated individuals in high-income economies have a strong propensity to choose renewable and ecologically beneficial forms of energy. Further, as the population increases, the population density increases, which makes clusters inside densely populated areas. As a result, the residential areas, offices, educational institutes, shopping plazas, restaurants, and hospitals come closer to each other, thus reducing the travelling distance and promoting walking, cycling, and public transport, lowering fossil fuel requirements. Consequently, population growth no longer adversely impacts environmental sustainability in these nations. This result aligns with the findings of Ganda [89].

Finally, the causality results confirm that any change in the ecological footprint will impact conventional economic growth and vice versa in both groups of countries. Similarly, the ecological footprint causes a change in green growth and vice versa in emerging and developed economies. If we see these results together with the long-run results, we suggest that green growth is the way forward for the world’s sustainable future for both developed and developing economies. Moreover, findings suggest that ICT, energy consumption, natural resources, eco-innovations, and population are important elements for causing a change in the ecological footprint in advanced economies. Nevertheless, improvement in environmental quality has implications for ICT, natural resources, energy consumption, and population in these economies. In the context of developing economies, a shock to ecological footprint cause changes in ICT, energy consumption, and population, and the same is true for the opposite scenario. In contrast, any change in ecological footprint for emerging economies will have a consequence for natural resources and eco-innovations but not valid for vice versa.

## Conclusion and policy implications

Rapidly growing industrialization and economic growth have accelerated the consumption and extraction of natural resources in group-7 and emerging-7 economies. However, green growth, environmental innovation, and ICT innovations are considered fundamental determinants that can be adopted to control ecological footprint. Thus, our study explores the effect of green growth, ICT, environment innovation, and natural resources on the ecological footprint from 1995–2020 for G-7 and E-7 economies. To test this objective, we have used the CS-ARDL and DCCE to confirm the results of baseline regression. The empirical findings under the CS-ARDL model reveal that green growth negatively influences the ecological footprint in emerging economies in the long run. However, natural resources enhance the ecological footprint in emerging economies. ICT and environmental innovation tend to reduce the ecological footprint in emerging economies in the long run. In the case of developed economies, CS-ARDL findings conclude that green growth, ICT, natural resources, and environmental innovation reduce the ecological footprint in the long run. The CS-ARDL findings are reconfirmed by employing the DCCE model. The empirical findings under the DCCE model reveal that green growth tends to reduce the ecological footprint in emerging economies in the long run. In contrast, natural resources tend to increase the ecological footprint in emerging economies. However, ICT and environmental innovation significantly reduce the ecological footprint in emerging economies in the long run. In the case of developed economies, DCCE findings conclude that green growth and environmental innovation reduce the ecological footprint in the long run. ICT and natural resources produce an insignificant impact on the ecological footprint in developed economies in the long run.

Our research made significant policy recommendations for developing and industrialized nations based on these findings. First, laws that encourage the protection of natural assets must be put into effect. To achieve green sustainability, natural resources should be moved into production. Second, if used correctly and successfully for optimal consumption that aids in reducing ecological impact, environmental innovation may provide additional benefits. Authorities should confirm the effectiveness of the deployment of eco-innovations. Finally, carbon taxes and the promotion of eco-friendly technology are necessary to promote sustained green development. In this context, fostering green growth and technical innovation is important. Finally, ICT growth should be encouraged since it lowers energy use and improves environmental performance. ICT-driven development that aids in the fight against air pollution and protects the sustainability of energy supplies must be encouraged.

Our study contains some limitations. The present study highlighted the impact of green growth, ICT, eco-innovation, and natural resources on the ecological footprint of emerging economies and developed economies at the aggregate level. In order to adopt more sensible policy recommendations for each country independently, future research may investigate this connection at a nation-wise disaggregated level. Future research might include interactive concepts into the framework for eco-innovation and natural resources. Just internet usage has been used in the current research to quantify ICT. In the next investigation, other ICT proxy metrics may be utilized. Finally, future research for developing and established economies might also take into account the impact of digital commerce.

## Supporting information

S1 Dataset (XLSX)
